# Anaemia, ethnicity, and cancer incidence: a retrospective cohort study in primary care

**DOI:** 10.3399/BJGP.2024.0762

**Published:** 2025-08-27

**Authors:** Liz Down, Melissa Barlow, Sarah ER Bailey, Luke TA Mounce, Samuel WD Merriel, Jessica Watson, Ge Chen, Tanimola Martins

**Affiliations:** 1 Department of Health and Community Sciences, University of Exeter, Exeter, UK; 2 Centre for Primary Care & Health Services Research, University of Manchester, Manchester, UK; 3 Centre for Academic Primary Care, Population Health Sciences, Bristol Medical School, University of Bristol, Bristol, UK

**Keywords:** anaemia, diagnosis, erythrocyte indices, ethnicity, ferritins, neoplasms, primary health care

## Abstract

**Background:**

Haemoglobin, mean corpuscular volume (MCV), and rates of iron-deficiency anaemia (IDA) are used in primary care to investigate possible cancer symptoms, especially for gastrointestinal cancers. Underlying ethnic differences in typical test results could lead to inequalities in the diagnosis of cancer in primary care.

**Aim:**

To investigate the distribution of low haemoglobin, low MCV, and IDA, and the rate of cancer diagnosis in patients with abnormal results, by ethnic group.

**Design and setting:**

A retrospective cohort study using routine data collected in primary care in England was undertaken. Included patients had blood tests between 2010 and 2017, and were aged ≥40 years with no prior cancer diagnosis.

**Method:**

Multilevel logistic regression was used to investigate the relationship between blood test results and cancer risk for patients in different ethnic groups.

**Results:**

Low haemoglobin, low MCV, and IDA were effective in identifying patients with increased cancer risk, particularly for gastrointestinal cancers. MCV was found to be a stronger cancer indicator for White patients (diagnostic odds ratio [OR] 3.84; 95% confidence interval [CI] = 3.72 to 3.96) than for Asian (OR 1.86; 95% CI = 1.64 to 2.10) or Black patients (OR 1.75; 95% CI = 1.54 to 1.99).

**Conclusion:**

There are some small differences in cancer risk for patients with abnormal test results, when considering patient ethnic group, especially for MCV. This is likely to be a consequence of the underlying difference in typical MCV values for patients from different ethnic groups. Further investigation is required to understand the aetiology of these differences in order to disentangle any effects on outcomes for patients with cancer.

## How this fits in

Previous research has shown that blood test results vary in healthy populations of different ethnic groups, with White patients typically having higher haemoglobin and mean corpuscular volume (MCV) levels, and lower levels of iron-deficiency anaemia (IDA) than patients from other ethnic groups. Little information was available, however, on why those differences are observed and the impact that this could have on cancer diagnosis rates. This study found that low MCV was a better indicator of cancer risk for White patients than for those in other ethnic groups, which may reflect the large difference in typical MCV values between the ethnic groups.

## Introduction

Timely diagnosis of cancer is crucial to improve patient outcomes and survival. Around 60% of patients in England are diagnosed after presenting to primary care with cancer symptoms.^1^

In England and Wales, there is guidance available to primary care clinicians for referring patients for cancer investigations, which is dependent on blood test results such as anaemia (Box 1).^2^ Several studies have identified a link between low haemoglobin levels, low mean corpuscular volume (MCV), or iron-deficiency anaemia (IDA) and increased gastrointestinal cancer risk.^3–8^

Box 1. **Summary of cancer referral guidelines for patients with anaemia in England and Wales^a^**

- Patients aged ≥55 years with low haemoglobin and upper abdominal pain should be investigated for oesophageal and stomach cancer.
- Women aged ≥55 years with low haemoglobin and visible haematuria should be investigated for endometrial cancer.
- Patients with iron-deficiency anaemia should be offered faecal immunochemical testing (FIT) for potential colorectal cancer.
- Patients aged ≥60 years with non-iron-deficiency anaemia should be offered FIT testing for potential colorectal cancer.

^*a*^ *From the National Institute for Health and Care Excellence guidelines for recognition and referral of suspected cancer* ^2^

Asian and Black patients tend to have, on average, lower haemoglobin and MCV levels than White patients,^9^ while in one study serum ferritin was highest for East Asians, with people categorised as Black or Hispanic having intermediate levels, and people from White and South Asian ethnic groups having the lowest levels.^10^ These blood measures may be affected by conditions such as sickle cell or thalassaemia. These are genetic conditions typically seen in people with African, Caribbean, Mediterranean, Asian, South American, or Middle Eastern heritage, where a particular haemoglobin variant is present, which can cause low haemoglobin or low MCV.^11,12^

The reasons for ethnicity-based differences in blood test results are complex and not fully understood, which can leave clinicians with a conundrum in trying to interpret an abnormal result. In the UK, blood test reference ranges and referral advice do not differ based on patient ethnicity, as there is limited research evidence available on this topic. In fact, there are arguments against ethnicity-based reference ranges, which have the potential to exacerbate health inequalities.^13,14^ This study aimed to investigate the distribution of blood test results in different ethnic groups, and the rate of cancer diagnosis in patients with results outside of the standard reference ranges, by ethnic group.

## Method

### Data source

The dataset used for this project consisted of an English primary care cohort, with additional linked data from secondary care and cancer registrations. The data were provided by the Clinical Practice Research Datalink (CPRD), which manages and links various UK health datasets. The dataset consisted of CPRD Aurum primary care data,^15^ linked to cancer registry data (National Cancer Registration and Analysis Service; NCRAS),^16^ and secondary care data (Hospital Episode Statistics Admitted Patient Care; HES APC)^17^ relating to patient ethnicity.

The cohort for this analysis was based on patients who had blood tests between 2010 and 2017. Included patients were aged ≥40 years at the time of the blood test, with no prior cancer diagnosis (except non-melanoma skin cancer), and a record of ethnicity.

### Outcome measures

The outcome measure for this analysis was a record of a cancer diagnosis within 1 year of the index test date. Two main analyses were carried out, the first assessing cancer incidence at any cancer site, and the second examining incidence of cancer at any site diagnosed at an advanced stage (tumour, node, and metastasis [TNM] stage T3 or T4, or M1).

Secondary outcomes were diagnosis with site-specific cancer within 1 year after the index test date. Cancer sites considered were oesophago-gastric, colorectal, and uterine, as these are the sites linked to haemoglobin and IDA in the National Institute for Health and Care Excellence (NICE) guidance for management of suspected cancer in England and Wales.^2^

### Main exposure measures

Using UK guidelines, low haemoglobin was defined as less than 130 g/l for men, and less than 120 g/l for women.^18^ Upper limits of normal haemoglobin were assessed from the dataset to be 180 g/l for men, and 160 g/l for women. Normal MCV was defined as a measurement of between 80 fl and 100 fl, after checking typical ranges applied within the dataset. Patients with high MCV or high haemoglobin were excluded from the corresponding analysis as the focus was on normal versus low results. Patients with ferritin below 30 µg/l, and a low haemoglobin result in the 30 days before the ferritin test were classified as having IDA.^18^ The index blood test used for analysis was the first one for each patient carried out between 2010 and 2017.

Patient ethnicity was derived from CPRD Aurum data where possible, with the addition of HES APC data where no ethnicity was available from the primary care data, using an established methodology.^19–22^ See Supplementary Information S1 for further details. Ethnicity was classified into one of five groups in line with the UK census: White (British, English, Welsh, Scottish, Northern Irish, any other White background), Asian (Indian, Pakistani, Bangladeshi, Chinese, any other Asian background), Black (African, Caribbean, any other Black background), Mixed (White and Black Caribbean, White and Black African, White and Asian, any other Mixed background), and Other (Arab, any other ethnic group).^23^

### Other covariates

Demographic covariates included patient sex, age group, deprivation quintile (Index of Multiple Deprivation [IMD] 2015),^24^ smoking status, body mass index (BMI) category, comorbidity tertile (Cambridge Multimorbidity Score; CMS),^25^ and the presence of haemoglobinopathy. An indicator was generated to denote patients with a record of common haemoglobinopathies, which identified patients with a record of sickle cell, thalassaemia variants, or unspecified haemoglobinopathy. The haemoglobinopathy flag applied to patients with any of these conditions, and carriers. Detailed information on variables generated can be found in Supplementary Information S2.

### Statistical analysis

Summary statistics were used to describe the cohort. Multilevel logistic regression, clustering patients within general practices, was used to investigate the relationship between blood test results and cancer risk for patients in each ethnic group, adjusted for the covariates described above. Odds ratios (ORs) comparing probability of cancer in those with and without an abnormal blood test result for each ethnicity were obtained through inclusion of an interaction term. The marginal distributions of the models were used to obtain adjusted cancer incidences.

Analyses were conducted using Stata/MP (version 18.0). Plots were generated using R 4.3.3, 'Angel Food Cake'. Results were reported in accordance with the ‘Reporting of studies conducted using observational routinely collected health data’ (RECORD) statement.^26^

### Patient and public involvement

The study was discussed with a public collaborator group specifically recruited for this purpose, ensuring representation from the three main ethnic groups analysed in this study (White, Asian, and Black). Conversations focused on the complexities of cancer diagnosis, including the nature of symptoms, blood tests, and the influence of health literacy, socioeconomic deprivation, and barriers to accessing care. These insights have shaped the interpretation of our findings.

## Results

### Cohort features

**Figure 1.**
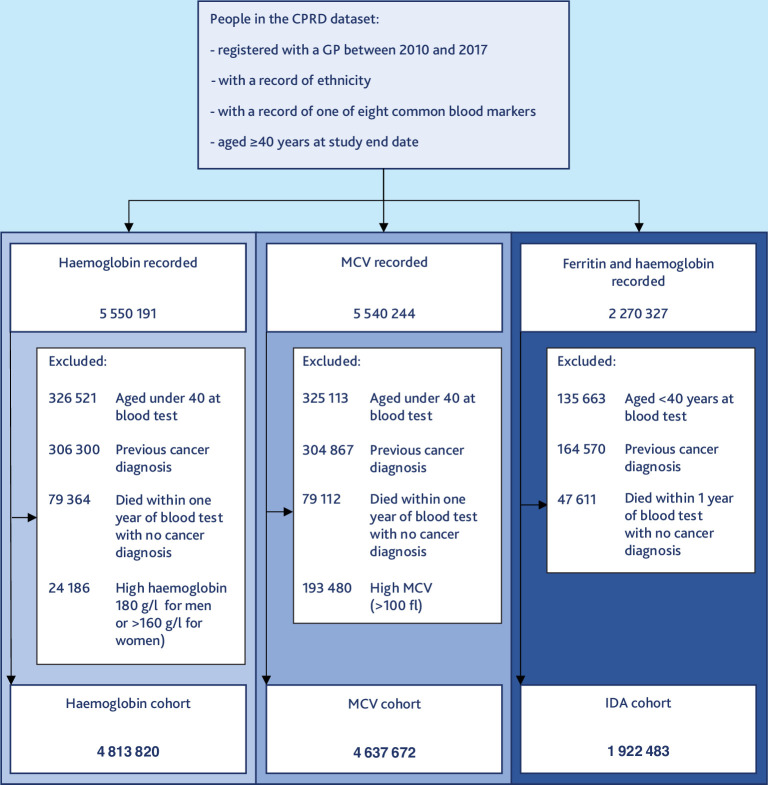
Cohort selection. CPRD = Clinical Practice Research Datalink. IDA = iron-deficiency anaemia. MCV = mean corpuscular volume.

The cohort included 4 813 820 patients with a haemoglobin result, 4 637 672 patients with an MCV result, and 1 922 483 patients with a ferritin result and corresponding haemoglobin result for calculation of IDA (Figure 1). In the haemoglobin cohort, 88% of patients were White, 7% were Asian, 4% were Black, 1% were in the Mixed group, and 1% were in the Other group (Table 1). The ethnic groups showed some general differences, with White patients likely to be older, living in a less deprived area, having higher morbidity burden, and more likely to have a record of a history of smoking (Supplementary Table S1). Black patients were most likely to be recorded as overweight or obese than the other groups. Rates of haemoglobinopathy were highest in the Black group (7%) and lowest in the White group (0%).

**Table 1.** Patient characteristics (haemoglobin cohort)

	Number of patients	Age (years)	Patients with a haemoglobinopathy	Patients in most deprived quintile	Patients diagnosed with any cancer within 1 year of test
	*n* (%)	Median (SD)	*n* (%)	*n* (%)	*n* (%)
White	4 211 361 (87.5)	58 (13.9)	7720 (0.2)	659 687 (15.7)	79 355 (1.9)
Asian	314 710 (6.5)	50 (11.7)	10 516 (3.3)	80 097 (25.5)	1921 (0.6)
Black	194 075 (4.0)	49 (11.5)	14 204 (7.3)	85 277 (43.9)	1864 (1.0)
Mixed	45 306 (0.9)	48 (11.2)	1948 (4.3)	12 784 (28.2)	416 (0.9)
Other	48 368 (1.0)	49 (11.6)	1291 (2.7)	13 671 (28.3)	476 (1.0)
Total	4 813 820	57 (13.8)	35 679 (0.7)	851 516 (17.7)	84 032 (1.7)

### Blood test results

Haemoglobin, MCV, and ferritin were found to have different typical values for the different ethnic groups (Table 2).

**Table 2.** Blood test result distribution by ethnic group^a^

	Median test result (interquartile range)			Per cent of patients % (number) with:		
	Haemoglobin (g/l)	MCV (fl)	Ferritin (µg/l)	Low haemoglobin	Low MCV	IDA
**White**	139 (130–149)	91.1 (88–94.3)	77 (34–152)	11.3 (476 529)	2.4 (103 497)	10.4 (168 274)
**Asian**	135 (125–147)	86.9 (82.8–90.4)	48.9 (21–103)	19.3 (60 894)	14.6 (46 065)	17.0 (28 963)
**Black**	133 (123–143)	87.7 (83.1–91.7)	72 (30–143)	22.9 (44 493)	14.3 (27 637)	14.5 (14 300)
**Mixed**	136 (126–147)	88.7 (84.5–92.4)	68 (29–138)	15.9 (7217)	10.4 (4728)	12.6 (2612)
**Other**	139 (129–149)	88.7 (85– 92)	66 (27–134)	12.8 (6218)	9.6 (4639)	11.8 (2545)

IDA = iron-deficiency anaemia. MCV = mean corpuscular volume.

a Blood test distribution values are based on the entire eligible patient group for each blood result, including those patients with raised haemoglobin or raised MCV.

Haemoglobin values were highest for White and Other patients, while MCV was highest for White patients. Also, 11.3% of White patients had low haemoglobin, compared with 19.3% of Asian patients, 22.9% of Black patients, 15.9% of patients in the Mixed group, and 12.8% of patients in the Other group. Low MCV was found in 2.4% of White patients, 14.6% of Asian patients, 14.3% of Black patients, 10.4% of patients in the Mixed group, and 9.6% of patients in the Other group.

Ferritin was found to be lowest for Asian patients with a median of 48.9 µg/l compared with between 66 µg/l and 77 µg/l for the other ethnic groups, which is reflected in higher rates of IDA for Asian patients at 17.0%, compared with 10.4% for White patients and 14.5% for Black patients.

Outside of ethnicity, sex, age, and the presence of haemoglobinopathy also had a substantial impact on haemoglobin, MCV, ferritin, and IDA status (see Supplementary Table S2).

Blood test distribution values are based on the entire eligible patient group for each blood result, including those patients with raised haemoglobin or raised MCV.

### Test results and cancer risk

As the number of patients in the Mixed and Other groups was low, estimates generated for these groups were accompanied by very large confidence intervals (CIs), so are presented in Supplementary Tables S3–S5 only.

**Figure 2.**
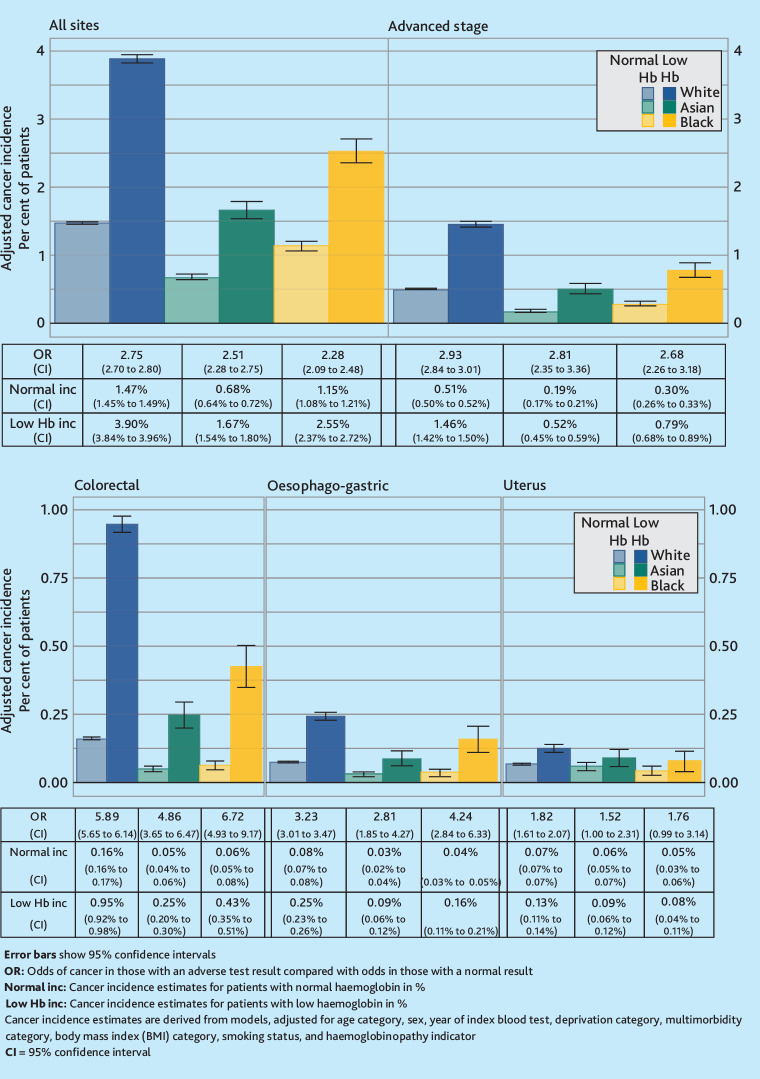
Cancer incidence by ethnicity and haemoglobin status.

**Figure 3. fig3:**
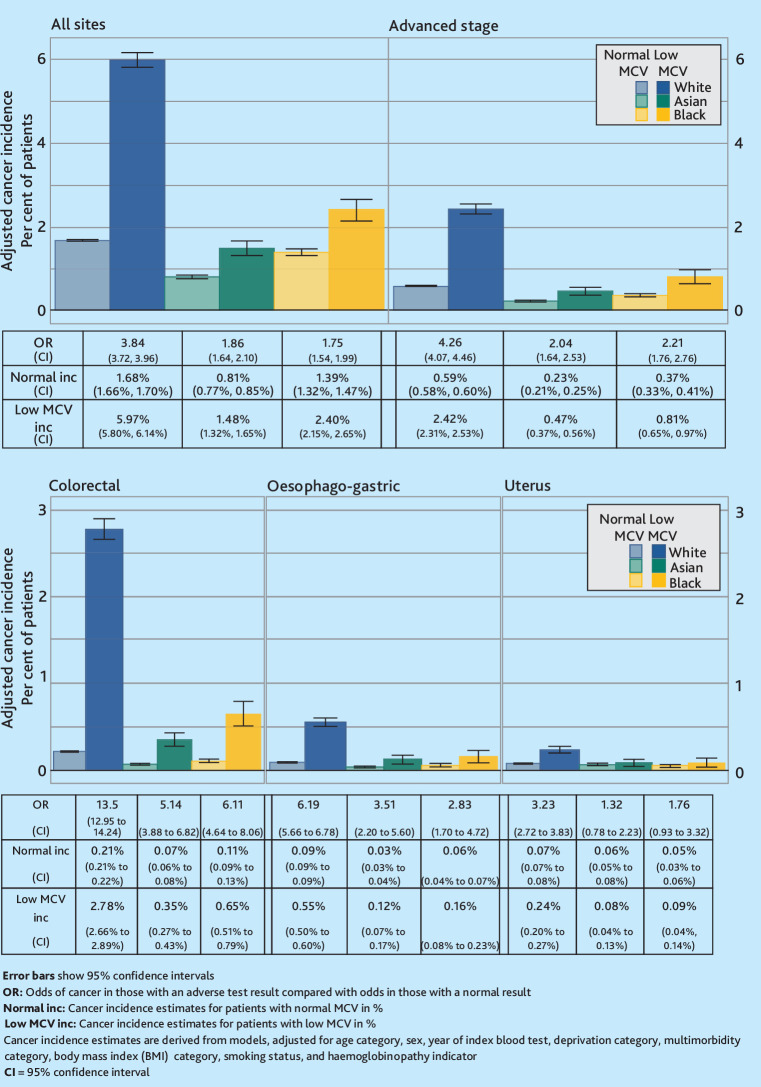
Cancer incidence by ethnicity and mean corpuscular volume (MCV) status.

**Figure 4.**
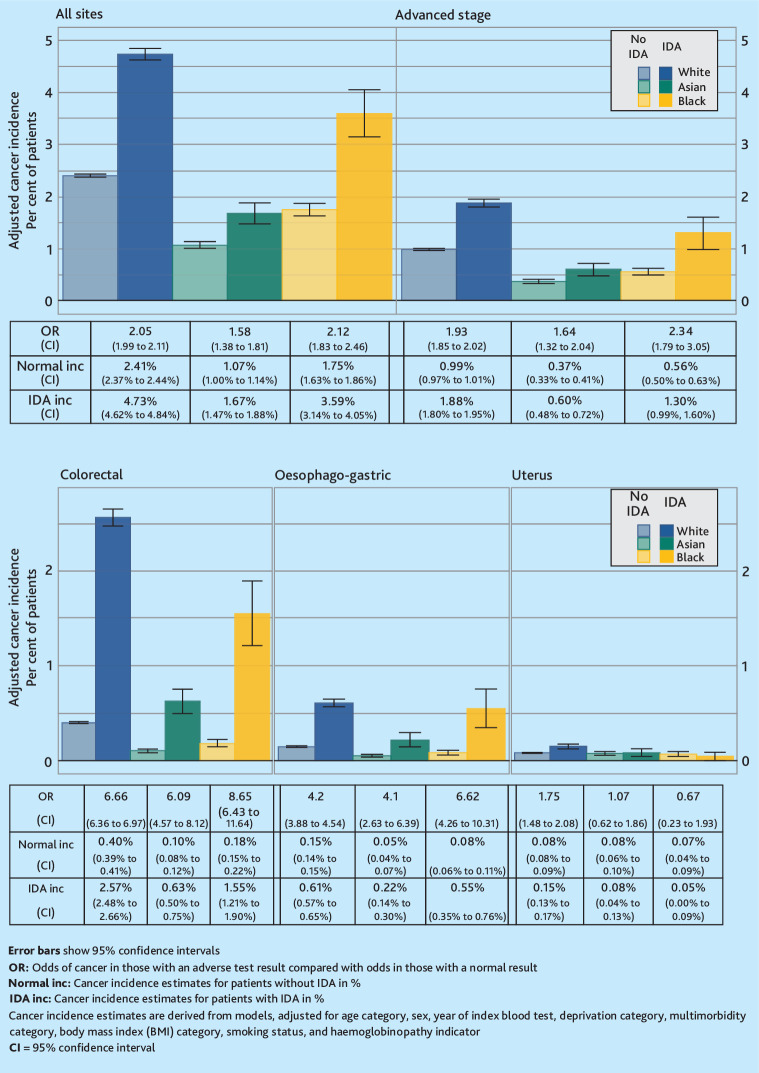
Cancer incidence by ethnicity and iron-deficiency anaemia (IDA) status.

Cancer incidence in the year following a low haemoglobin, low MCV, or IDA observation differed between the ethnic groups, for all-site cancer, cancer diagnosed at an advanced stage, and colorectal cancer (Figures 2 [Fig fig3]-4). For example, all-site cancer incidence in the year after a low MCV result for White patients was 6.0% (95% CI = 5.8% to 6.1%), for Asian patients 1.5% (95% CI = 1.3% to 1.7%), and for Black patients 2.4% (95% CI = 2.2% to 2.7%) Figure 3. Additionally, Asian patients with IDA had a lower oesophago-gastric cancer incidence in the year following an IDA observation than White or Black patients Figure 4.

For the assessment of all-site cancer risk, advanced cancer, colorectal cancer, and oesophago-gastric cancer, the ORs for cancer risk in patients with abnormal blood results compared with those with normal results were all above 1 (Figures 2 [Fig fig3]-4). However, the CI for uterine cancer ORs for some ethnic groups crossed below 1.

For low haemoglobin and IDA, the only ethnic difference in ORs was for all-site cancer risk (Figures 2 and 4). In the haemoglobin analysis, White patients had the highest OR for all-site cancer risk at 2.75, compared with Black patients at 2.28, while the CI for Asian patients crossed both of these ORs. For IDA, Asian patients had the lowest OR at 1.58, compared with White patients (2.05) and Black patients (2.12).

Each of the analyses of low MCV and cancer risk demonstrated a higher OR for White patients than for patients in other groups (Figure 3). For example, the all-site cancer OR was 3.84 (95 % CI = 3.72 to 3.96) for White patients, 1.86 (95% CI = 1.64 to 2.10) for Asian patients, and 1.75 (95% CI = 1.54 to 1.99) for Black patients.

## Discussion

### Summary

Low MCV was a much stronger indicator of cancer risk for White patients than the other groups. Low haemoglobin was found to be a stronger indicator of all-site cancer risk for White patients than for Black patients, while IDA was a weaker indicator of all-site cancer risk for Asian patients than those in other groups.

There were differences in the proportion of patients with an abnormal test result by ethnicity, which was most stark for MCV.

For all analyses except uterine cancer, cancer incidence differed between the three ethnic groups studied. This reflects ethnicity-specific cancer rates typically observed in UK cohorts and was not examined in detail.

Low haemoglobin and IDA showed a similar pattern of results, with similar degrees of association between test result and cancer risk for most of the analyses carried out, with the exception of the all-site cancer risk, where the association was strongest in the White group for haemoglobin, and for White and Black patients for IDA.

Prediction of cancer risk by low MCV result differed by ethnic group: the ORs for White patients were around double those of Asian or Black patients. Despite adjustment for available demographic and health characteristics in the current authors’ model, there are likely to be other differences between ethnic groups linked to socioeconomic determinants of health and factors relating to access to the healthcare system. Additionally, the very low rate of low MCV in White patients compared with Asian patients and Black patients may have had a substantial impact on the analysis.

The ORs for uterine cancer were lower than the other core cancer sites. The analyses for each of the three blood tests generated ORs for White patients above 1, but the CIs for Black and Asian patients crossed below 1. This could be a reflection of the lower numbers of patients in the Black and Asian groups, combined with these tests being less effective at identifying uterine cancer compared with gastrointestinal cancers.

### Strengths and limitations

This analysis was based on a large dataset covering approximately 20% of the UK population^27^ and a timeframe of 8 years. The primary care dataset is linked to NCRAS, the definitive record of cancer diagnoses in England. Ethnicity recording in the combined CPRD Aurum and HES dataset was available for more than 90% of patients, and is representative of the general population of England.^28,29^

This project used combined ethnic grouping for analyses, both because this ensured that patients with ethnicity recorded at any level of detail could be included, and also provide large enough groups to encompass a sufficient number of cancer events. It is recognised that this may obscure differences between ethnic sub-groups. Furthermore, it was not possible to draw conclusions from the analyses involving the Mixed and Other ethnic groups owing to the inherent heterogeneity and relatively low numbers.

The reason for blood tests was not assessed, so it is not known which blood tests were carried out because of concern about the possibility of cancer. This is reflected in the low cancer incidence rates observed. It is possible that there are ethnic biases in the process of GP attendance and blood test request, but this has not been investigated in this study.

These findings relating to uterine cancer were mixed, with only White patients having a clearly increased risk of cancer after an abnormal test result. Uterine cancer was included in this analysis because of its occurrence in the guidance^2^ as a potential cause of low haemoglobin levels, although in the guidance further investigation is only indicated for patients aged ≥55 years with visible haematuria.

### Comparison with existing literature

The association between anaemia and cancer risk or risk of cancer at an advanced stage has been previously reported in other studies.^7,30–33^ More specifically, this work found links with gastrointestinal cancer risk^3–7,30,31,34–36^ as expected. The use of these markers to identify patients at increased risk of uterine cancer was more tenuous in the current study, reflected in the sparse supporting literature, although Hung *et al* ^31^ did identify a link between IDA and uterine cancer.

The literature concerning haemoglobin, MCV or ferritin results, ethnicity, and cancer risk is very limited. One study was identified,^37^ reporting that anaemia was more prevalent in Black patients than in White patients, both in those with gastric cancer and those without, although the relative difference was not quantified. Therefore, in descriptive terms the data presented here are consistent with this study, but it is not possible to compare the outcomes.

### Implications for research and practice

Differences were observed in typical blood test measurements in patients from different ethnic groups. The reasons for these differences are not well understood, but may be the result of a complex array of environmental and social factors, in addition to some genetically determined effects.^10,38–40^

Despite this, the available evidence does not support using tailored reference ranges for patients from different ethnic groups, both because the causes of this variation are unclear, and because there is little research into the health effects of these differential blood test results.

The outcomes of this analysis suggest that it is more difficult to identify Black or Asian patients with raised cancer risk from abnormal blood results alone, and primary care cancer risk stratification needs to incorporate additional data.

These findings suggest that, for these cancer sites, observed ethnic inequalities in diagnostic routes, intervals, or stages^19,41,42^ may not be explained by variations in the performance of blood tests. Studies on the determinants of ethnic inequalities in these sites may focus on symptom reporting and acceptance of investigation in primary care.

Further scrutiny of test results and the indications for their use in primary care may be helpful to improve the evidence base in this area. For example, treating the test result as a continuous variable may add nuance to this picture. Trends in tests over time might also be useful for detection of cancer in primary care. Accounting for ethnicity might improve these models.^43^

In order to interpret the findings of this project, it would be valuable to further investigate the reasons behind the large disparity in MCV levels between ethnic groups, and to understand its clinical implications. Including other factors in future studies, such as detailed genetic, environmental, dietary, and socioeconomic data, could improve the understanding of the reasons for variation in blood test results.

In conclusion, there were notable differences in commonly used haematological test values across different ethnic groups, particularly in MCV levels, where Black and Asian patients were more likely to present with low MCV compared with White patients. The diagnostic ORs for haemoglobin and IDA showed some ethnic differences for all-site cancer, but demonstrate minimal variation in cancer risk prediction for advanced cancer or site-specific analysis. MCV appears to be a more effective cancer indicator for White patients than for others for all sites assessed, although the underlying reasons and appropriate clinical responses remain unclear.

## Data

The data were provided under licence from CPRD; therefore it is not possible to share this dataset.
